# Intraperitoneal prophylaxis with CpG oligodeoxynucleotides protects neutropenic mice against intracerebral *Escherichia coli* K1 infection

**DOI:** 10.1186/1742-2094-11-14

**Published:** 2014-01-23

**Authors:** Sandra Ribes, Tanja Meister, Martina Ott, Sandra Redlich, Hana Janova, Uwe-Karsten Hanisch, Stefan Nessler, Roland Nau

**Affiliations:** 1 Institute of Neuropathology, University Medical Center Göttingen, Göttingen, Germany; 2 Department of Geriatrics, Evangelisches Krankenhaus Göttingen-Weende, Göttingen, Germany

**Keywords:** CpG ODN, CNS infection, IL-12/IL-23p40, MIP-1α, IFN-γ, *Escherichia coli*, Neutropenia

## Abstract

**Background:**

Prophylaxis with unmethylated cytosine phosphate guanidine (CpG) oligodeoxynucleotides (ODN) protects against several systemic experimental infections. *Escherichia coli* is a major cause of Gram-negative neonatal bacterial meningitis and also causes meningitis and meningoencephalitis in older and immunocompromised patients.

**Methods:**

Wild-type (wt) and Toll-like receptor 9 (TLR9)-deficient mice were rendered neutropenic by intraperitoneal administration of the anti-Ly-6G monoclonal antibody. Immunocompetent and neutropenic mice received intraperitoneal CpG ODN or vehicle 72 h prior to induction of *E. coli* K1 meningoencephalitis.

**Results:**

Pre-treatment with CpG ODN significantly increased survival of neutropenic wt mice from 33% to 75% (*P* = 0.0003) but did not protect neutropenic TLR9^-/-^ mice. The protective effect of CpG ODN was associated with an enhanced production of interleukin (IL)-12/IL-23p40 with sustained increased levels in serum and spleen at least for 17 days after conditioning compared to buffer-treated animals. CpG-treated neutropenic wt mice showed reduced bacterial concentrations and increased recruitment of Ly6C^high^CCR2^+^ monocytes in brain and spleen 42 h after infection. The levels of macrophage inflammatory protein 1α (MIP-1α) and interferon gamma (IFN-γ) in spleen were higher 42 h after infection in CpG-treated compared to buffer-treated neutropenic animals. In immunocompetent mice, prophylaxis with CpG ODN did not significantly increase survival compared to the buffer group (60% *vs.* 45%, *P* = 0.2).

**Conclusions:**

These findings suggest that systemic administration of CpG ODN may help to prevent bacterial CNS infections in immunocompromised individuals.

## Background

Immunocompromised individuals, also the elderly, have an increased risk of experiencing systemic and central nervous system (CNS) infections [1,2]. In neutropenic patients, the causative agents of meningitis often are enteric Gram-negative bacilli such as *Escherichia coli* that live in the patient’s own digestive tract [3]. The ability of bacteria to escape the host defense and achieve the threshold of bacteremia necessary for subsequent invasion of the brain is probably higher in immunocompromised individuals than in immunocompetent adults thus explaining the differences in the occurrence of *E. coli* K1 meningitis [4]. Head trauma, neurosurgical interventions, or sepsis are other risk factors for the development of *E. coli* meningitis in adults either as a consequence of the impairment of the local host defense or subsequent to direct inoculation of bacteria into the CNS [5,6]. In immunodeficient and older persons the efficacy of current vaccines is low [7]. Moreover, immunization efficacy probably decreases with complex vaccination regimes against multiple pathogens. Vaccination against the majority of pathogens which may cause an infection in immunocompromised patients is an unrealistic goal. Thus, it seems rational to pursue a concept of pattern-specific stimulation of the innate immune system with the goal of increasing resistance to infections by several pathogens in the immunocompromised host.

Bacterial DNA containing unmethylated cytosine-guanidine motifs linked by a phosphodiester (p) group (CpG) activates mammalian lymphocytes and macrophages to produce cytokines including tumor necrosis factor (TNF)-α, interleukin (IL)-6, IL-12, and interferon gamma (IFN-γ), which are crucial for the immune response to bacterial infections [8]. CpG oligodeoxynucleotides (ODN) are short single-stranded DNA molecules which contain unmethylated CpG motifs and mimic bacterial DNA with immunostimulatory properties [9]. CpG-containing motifs are considered pathogen-associated molecular patterns (PAMPs) and are recognized by the pattern recognition receptor (PRR) Toll-like receptor 9 (TLR9) [10]. We recently showed that stimulation of primary murine microglial cells with CpG ODN 1668 increases phagocytosis and intracellular killing of *E. coli* K1, an important pathogen for meningitis and meningoencephalitis [11,12]. In previous studies with experimental animals, CpG ODN pre-treatment conferred protection against a variety of bloodstream and other extracerebral bacterial infections [13-20].

In this study, we investigated the protective properties of CpG ODN 1668 pre-treatment in immunocompetent mice as well as immunocompromised animals which were depleted of granulocytes. To mimic infections after cerebral/spinal trauma or surgery, murine meningoencephalitis was induced by direct injection of *E. coli* K1 into the CNS. Here, we report for the first time that CpG ODN induces protection against a primary bacterial CNS infection in neutropenic mice in a TLR9-dependent manner but not in immunocompetent animals. CpG prophylaxis promoted bacterial clearance which correlated with enhanced production of IL-12/IL-23p40, IFN-γ, and MIP-1α, and increased recruitment of Ly6C^high^CCR2^+^ monocytes at early infection.

## Methods

### Mice and monitoring

The animal experiments were approved by the Animal Care Committee of the University Hospital of Göttingen and by the *Niedersächsische Landesamt für Verbraucherschutz und Lebensmittelsicherheit* (*LAVES*), Braunschweig, Lower Saxony, Germany. C57Bl/6 wild-type (wt) (2 to 3 months old, weight 20 to 30 g, Charles River Laboratory) and TLR9-deficient animals on a C57Bl/6 background (2 to 5 months old, weight 20 to 32 g) were used in all experiments [10]. Animals were weighed and scored daily (0, no apparent behavioral abnormality; 1, moderate lethargy; 2, severe lethargy; 3, unable to walk; 4, dead) [21].

### CpG ODN

In this study, we used CpG ODN 1668 (5′ TCC ATG ACG TTC CTG ATG CT, molecular mass 6382.6 g/mol, TIB Molbiol, Berlin, Germany) which has potent immunostimulatory effects on primary cultures of microglial cells [11,12]. CpG ODN 1668 is a phosphorothioate (PTO) ODN that was dissolved in distilled water and stored at -80°C. CpG ODN was administered intraperitoneally (ip) 3 days prior infection at a dose of 100 μg per mouse in 200 μL phosphate buffered saline (PBS). The buffer group received the same amount (34 μL) of distilled water in 200 μL PBS. The use of a control CpG ODN 1668 (5′ TCC ATG AGC TTC CTG ATG CT) without immunostimulatory CpG motifs had no protective effect under the same experimental conditions (data not shown).

### Bacteria

The *E. coli* strain K1 (serotype O18:K1:H7) originally isolated from the CSF of a child with neonatal meningitis (gift of Dr. Gregor Zysk, Institute of Medical Microbiology, Düsseldorf, Germany) was used in all experimental infections [22]. Bacteria were grown overnight on blood agar plates, harvested in 0.9% saline and stored at -80°C. Frozen aliquots were used for the experiments and diluted with saline to the required bacterial concentration.

### Experimental design

**Figure 1:**
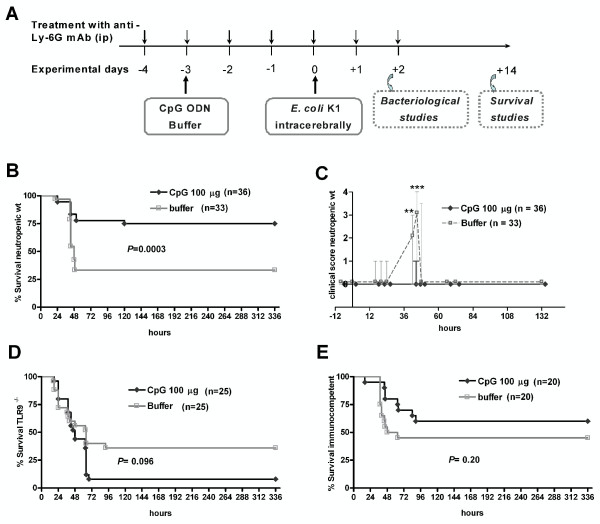
**CpG ODN protected neutropenic mice from *E. coli *meningoencephalitis. (A)** Experimental design and impact of CpG ODN pre-treatment (100 μg ip). **(B)** Survival and **(C)** clinical score of wt neutropenic mice. Survival of **(D)** TLR9^-/-^ neutropenic and **(E)** immunocompetent wt mice. Meningoencephalitis was induced by injection of 1 × 10^4^ CFU *E. coli* K1. Kaplan-Meier curves were compared by log-rank test. Differences in clinical scores between CpG- and buffer-treated mice were analyzed by Mann-Whitney U test (**P <0.01; ***P <0.001) and data are shown as median ± 25th/75th percentiles.

The experimental design with neutropenic mice is summarized in Figure 1A. Depletion of CD11b^+^Ly-6G^+^Ly-6C^int^ neutrophils was achieved by ip injection of 50 μg of anti-Ly6G monoclonal antibody (mAb, clone 1A8, BioXcell, West Lebanon, NH, USA) [22]. Anti-Ly6G mAb was administered daily starting 4 days before infection with a total of seven injections (from day -4 to day +2, infection performed at day 0). Meningoencephalitis was induced by injection of *E. coli* K1 into the superficial right frontal neocortex of the anesthetized animals. Neutropenic wt and TLR9^-/-^ mice were inoculated with 1 × 10^4^ colony forming units (CFU)/mouse while immunocompetent wt animals received 1 × 10^5^ CFU/mouse. In most of the survival experiments, animals were monitored over a 14-day period after infection but in one survival experiment, animals were observed for 2 months. In bacteriological studies, animals were sacrificed 42 h after infection.

### Sample processing

Serial blood samples were obtained by retro-orbital punction at different time points for cyto/chemokine measurement. Blood was collected, stored at 4°C for 30 min and then centrifuged at 3,000 × g for 10 min at 4°C. Serum was then transferred to another tube and stored at -20°C until performance of the different ELISAs. At end time point, animals were sacrificed by cervical dislocation. Blood was obtained by intracardiac punction. The whole brain and spleen were removed, and the cerebellum was dissected from the brain stem. Half of the spleen and half of the cerebellum were homogenized in 0.9% saline. Homogenates were serially diluted in 0.9% saline and plated on blood-agar plates (detection limit: 200 CFU/mL and 40 CFU/mL in cerebellar and spleen homogenates, respectively).

### Flow cytometry

Anesthetized animals were perfused transcardially with PBS 42 h after infection, and spleens and the right (inoculated) hemispheres were removed and processed. Brain hemispheres were separately digested and homogenized with collagenase D (2.5 mg/mL, Roche Diagnostics GmbH, Mannheim, Germany) and DNase I (2 mg/mL, Roche Diagnostics GmbH) using the gentle MACS dissociator (Miltenyi Biotec, Germany). The resultant homogenates were mechanically dissociated and passed through a 70-μm nylon cell strainer (BD Biosciences, Franklin Lakes, NJ, USA). Leukocytes were separated by a 37/70% Percoll gradient (GE Healthcare, Chalfont St Giles, Buckinghamshire, UK). Spleens were also separately passed through a 70-μm nylon strainer and processed as previously described [23]. Single cells were stained with the following antibodies: CD45 (30-F11), CD4 (RM4-5), CD27 (LG.3A10), CD11b (M1/70), and Ly6C (HK1.4) purchased from BioLegend (San Diego, CA, USA), CD3 (145-2C11), CD25 (PC61.5), CD19 (eBio1D3) NK1.1 (PK136), and FoxP3 (FJK-16 s) provided by eBioscience (San Diego, CA, USA), and Ly6G (1A8, BD Pharmigen, Franklin Lakes, NJ, USA) and CCR2 (FAB5538A, R&D Systems, Minneapolis, MN, USA). At least 50,000 events were acquired on a FACSCanto II (BD Biosciences) and analyzed using FlowJo software (version 8.8; Tree Star).

### Cyto-/chemokine measurements

Levels of IL-12/IL-23p40, IL12p70, IFN-γ, and macrophage inflammatory protein 1α (MIP-1α) were determined by DuoSet ELISA Development Kits (R&D Systems, Wiesbaden, Germany) according to the manufacturer’s instructions [24]. Cyto-/chemokines were measured in cerebellar and splenic homogenates. Additionally, IL-12/IL-23p40 was determined in serum samples collected at different time points (52 h and 5 h before infection as well as 42 h and 14 days after infection). The sensitivity was 7.5 pg/mL for all tested cyto-/chemokines. When measurements were below the level of detection, a value of 7.4 was taken for statistical analysis.

### Histological analysis

Paraffin-embedded, 2-μm coronal brain sections from neutropenic mice sacrificed 42 h after infections were analyzed. Chloroacetate esterase (CAE) stainings were performed to evaluate the degree of inflammation in three superficial meningeal regions and the hippocampal fissure. Stained sections were blinded and semi-quantitatively scored for the number of CAE-stained leukocytes in one high-power field (×40 objective) per region by a blinded investigator: no leukocytes (score 0), <10 leukocytes (score 1), 10-50 leukocytes (score 2), >50 leukocytes (score 3). For each animal, the scores of the individual fields were added and then divided by the number of scored regions.

### Statistical analysis

Survival was compared using the log-rank test. Differences between buffer- and CpG-treated groups were analyzed by the Mann-Whitney U test and Fisher’s exact test. Data are expressed as medians (25th/75th percentiles). The correlation between bacterial titers and cyto-/chemokine levels was analyzed using Spearman’s rank correlation coefficient. For all analyses, GraphPad Prism version 5 (GraphPad Software, San Diego, CA, USA) was used, and a *P* value <0.05 was considered statistically significant.

## Results

### CpG ODN protects neutropenic animals from *E. coli* meningoencephalitis in a TLR9-dependent manner

A single intraperitoneal (ip) injection of 100 μg CpG ODN 3 days before infection protected neutropenic wt mice against intracerebral infection with *E. coli* K1 (Figure 1B). Survival at 14 days after infection was 75% (27/36) in the CpG- *versus* 33% (11/33) in the buffer-treated group (*P* = 0.0003; log-rank test). Accordingly, buffer-treated animals exhibited more severe clinical symptoms at 42 h and 45 h after infection than CpG-treated mice (*P* ≤0.003; Mann-Whitney U test) (Figure 1C). Neutropenic TLR9^-/-^ mice were not protected from intracerebral infection with 1 × 10^4^ CFU *E. coli* K1 by pre-treatment with 100 μg CpG ODN (*P* = 0.10, log-rank test; survival 14 days after infection 8% (2/25, CpG group) *versus* 36% (9/25, buffer group) (Figure 1D).

### CpG ODN effect as a prophylactic agent against meningoencephalitis in immunocompetent animals

Mice with an intact granulocyte function were less susceptible to intracerebral *E. coli* K1 infection (LD_50_ = 6 × 10^4^ CFU) than mice which were depleted from circulating neutrophils by mAb 1A8 administration (LD_50_ = 5 × 10^3^ CFU). The protection conferred by a single ip dose of 100 μg CpG ODN was mild in immunocompetent animals and failed to reach statistical significance (Figure 1E; *P* = 0.2; log-rank test; survival 14 days after infection was 60% (12/20) in CpG ODN group *versus* 45% (9/20) in the buffer group). Although the differences also were not statistically significant (*P* = 0.09; Mann-Whitney U test), buffer-treated animals tended to show a higher clinical score than CpG-treated mice at the early phase of meningoencephalitis (42 h after infection).

### Intraperitoneal administration of CpG ODN decreases weight in uninfected wt mice but not in TLR9-deficient mice

A sensitive indicator of sickness behavior of uninfected neutropenic wt mice which received a single ip injection of 100 μg CpG ODN (*n* = 46) was loss of weight [25]. Twenty-four hours after CpG ODN administration the weight was 94.1% (92.9/95.2), whereas in immunocompromised control animals (*n* = 42) weight remained stable at 101.3% (100.0/102.2) (*P* <0.0001; Mann-Whitney U test). Uninfected immunocompetent mice (*n* = 20/group) also lost weight after a single injection of 100 μg CpG ODN: 24 h after CpG ODN administration the weight was 95.7% (94.3/96.7%), whereas immunocompetent buffer-treated animals did not lose weight (98.7% (97.0/101.3%)) (*P* <0.0001; Mann-Whitney U test). Within the following 3 days, CpG ODN-treated uninfected mice regained their pre-treatment weight. Conversely, the weight of TLR9 deficient mice (*n* = 25/group) was not influenced by the administration of CpG ODN (99.1% (97.6/100.4)).

### Pre-conditioning with CpG ODN decreases the bacterial concentrations in cerebellar and spleen homogenates in neutropenic wt mice at the early phase of infection

**Figure 2:**
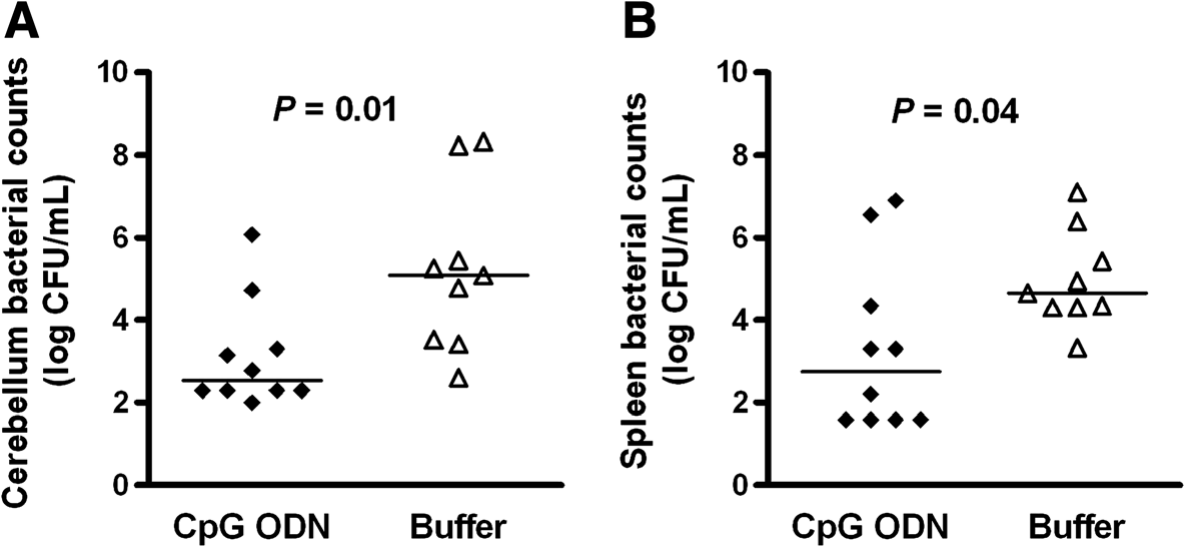
**CpG ODN pre-treatment decreased bacterial loads in the early phase of meningoencephalitis in neutropenic mice.** Bacterial concentrations in **(A)** cerebellum and **(B)** spleen were determined 42 h after intracerebral *E. coli* K1 infection (1 × 10^4^ CFU/mouse). *n* = 9-10 mice/group. Horizontal bars indicate median values. Statistical analysis was performed by Mann-Whitney U test.

Neutropenic wt mice pre-conditioned with 100 μg CpG ODN (*n* = 10) or treated with buffer solution (*n* = 9) were sacrificed 42 h after infection to determine the protective effect of CpG ODN. The bacterial density in cerebellum homogenates of CpG-treated mice was 2.54 (2.30/3.66) log_10_CFU/mL compared to 5.09 (3.47/6.84) log_10_CFU/mL in buffer-treated mice (*P* = 0.01; Mann-Whitney U test) (Figure 2A). The bacterial density in the spleen in CpG-treated mice was 2.75 (1.59/4.90) log_10_CFU/mL compared to 4.65 (4.30/5.92) log_10_CFU/mL in buffer-treated animals (*P* = 0.04; Mann-Whitney U test) (Figure 2B).

### CpG-treated neutropenic animals that survived the infection show a delay in the clearance of bacteria

**Figure 3:**
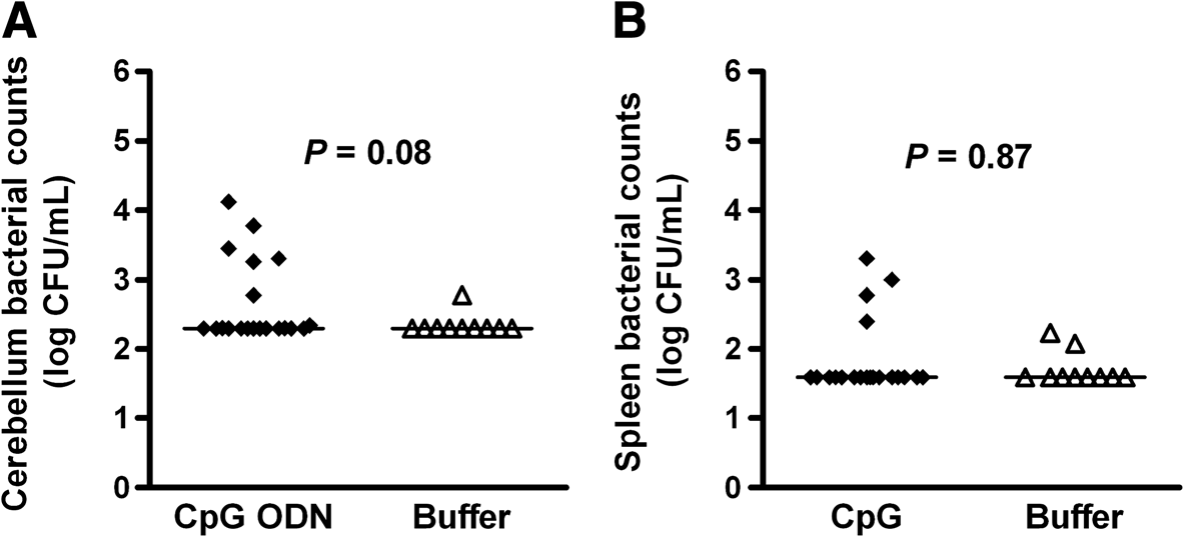
**CpG-treated neutropenic animals that survived the infection showed a delay in the clearance of bacteria.** Bacterial concentrations in **(A)** cerebellum and **(B)** spleen were determined 14 days after intracerebral *E. coli* K1 infection in those animals which survived the infection (*n* = 10-20 mice/group). Horizontal bars indicate median values. Statistical analysis was performed by Mann-Whitney U test.

After 14 days of infection, 45% (9/20) of the surviving neutropenic wt animals pre-conditioned with CpG ODN still showed positive bacterial cultures in cerebellum homogenates *versus* 10% (1/10) of the buffer-treated animals (Figure 3A, *P* = 0.08 by Mann-Whitney U test; *P* = 0.10 by Fisher’s exact test). Positive cultures in spleen homogenates were found in 20% (4/20) of CpG-treated and in 20% (2/10) of buffer-treated animals (Figure 3B). We therefore performed a survival experiment in which buffer and CpG-treated animals (*n* = 9 mice/group) were infected with 1 × 10^4^ CFU/mouse and monitored for 2 months. Two months after infection, the bacterial cultures in all surviving animals (7/9 in the CpG- and 1/9 in the buffer-treated groups) were negative (level of detection: 200 CFU/mL and 40 CFU/mL in cerebellum and spleen homogenates, respectively). In all survival experiments, no mortality occurred later than day 6 after infection. All surviving immunocompetent mice had negative bacterial cultures in tissue homogenates 14 days after infection.

### CpG ODN immunization increases the percentage of inflammatory monocytes in the infected CNS and spleens of neutropenic mice

Pre-treatment with CpG ODN can modify leukocyte populations in infected tissues [26]. We therefore analyzed distinct cell types in the spleens and brains (right hemisphere containing the site of injection) of neutropenic mice at 42 h after *E. coli* K1 infection by flow cytometry. The absolute and relative numbers of the different cell subsets found in the CNS are depicted in Table 1. Flow cytometry revealed absence of CD11b^+^Ly6G^+^Ly6C^int^ neutrophils in spleen and brain in all anti-Ly6G-treated mice (data not shown). The percentage of inflammatory monocytes (CD45^+^CD11b^+^Ly6C^high^CCR2^+^) among CD45^+^ leukocytes was significantly higher in CpG-treated mice (*P* = 0.035; Mann-Whitney U test) while the total number of inflammatory monocytes/animal did not quite reach statistical significance (*P* = 0.073; Mann-Whitney U test). The percentage of T cells (CD45^+^CD3^+^) and regulatory T cells (CD45^+^ CD4^+^CD3^+^CD25^+^FoxP3^+^) among CD45^+^ leukocytes significantly decreased in the CNS of CpG-treated animals (*P* ≤0.04; Mann-Whitney U test), while the percentage of NK cells (CD45^+^NK1.1^+^CD3^-^) remained unaffected. The numbers and percentages of CD45^+^CD11b^+^Ly6C^high^CCR2^-^, CD45^+^CD11b^+^Ly6C^int^CCR2^-^ monocytes and CD45^int^CD11b^int^Ly6C^-^ microglial cells were comparable between CpG ODN- and buffer-treated animals.

**Table 1:** Subpopulations of T cells in the CNS of CpG- and buffer-treated neutropenic mice 42 h after infection

	**Absolute no. of cells**^**a**^		**Percent among all CD45**^**+ **^**cells**^**a**^	
	**CpG**	**Buffer**	**CpG**	**Buffer**
CD45^+^ cells	237427 (122770/257348)	228016 (169274/314119)		
T cells (CD45^+^CD3^+^)	4274 (3560/4607)	4844 (3065/8602)	2 (1.8/2.9)^b^	3.2 (2.8/3.6)
Regulatory T cells CD45^+^CD4^+^CD3^+^CD25^+^FoxP3^+^	172 (122/307)	375 (167/489)	0.08 (0.06/0.13)^b^	0.2 (0.12/0.55)
NK cells CD45^+^NK1.1^+^CD3^-^	3631 (3138/7180)	2588 (1882/6996)	2.6 (1.6/3)	2.2 (1.5/3.6)
Inflammatory monocytes CD45^+^CD11b^+^Ly6C^high^CCR2^+^	66905 (46994/101306)	40683 (23199/55803)	39.4 (27.6/46.9)^b^	25.9 (23.3/28.9)
Monocytes CD45^+^CD11b^+^Ly6C^high^CCR2^-^	13798 (6089/22579)	13150 (7054/24206)	7 (4.9/11)	8.54 (5/11.7)
Monocytes CD45^+^CD11b^+^Ly6C^int^CCR2^-^	40918 (15076/69499)	39167 (19894/65079)	22 (15.9/27.51)	26.4 (17.35/34)
Microglia CD45^int^CD11b^int^Ly6C^-^	6392 (4150/27330)	5316 (2869/18381)	3.56 (2.6/10.62)	4.69 (2.48/7.71)

^a^Data are medians (25th/75th percentiles) determined by forward and side-scatter properties from the right hemisphere of animals (*n* = 6-7/group) of at least three separate experiments.

^b^Significantly different (*P* <0.05) *vs.* buffer-treated group (Mann-Whitney U test).

In the spleen, pre-treatment with CpG ODN significantly increased the percentage of inflammatory monocytes among all leukocytes (*P* = 0.009; Mann-Whitney U test) compared to buffer-treated animals. The percentages of T cells and regulatory T cells among all CD45^+^ cell population were significantly decreased in the spleens (*P* ≤0.009; Mann-Whitney U test) of infected CpG-treated mice compared to buffer-treated animals. The percentage of NK cells was not influenced by pre-treatment with CpG ODN.

### Cyto-/chemokine response to CpG ODN treatment in neutropenic infected mice

**Figure 4:**
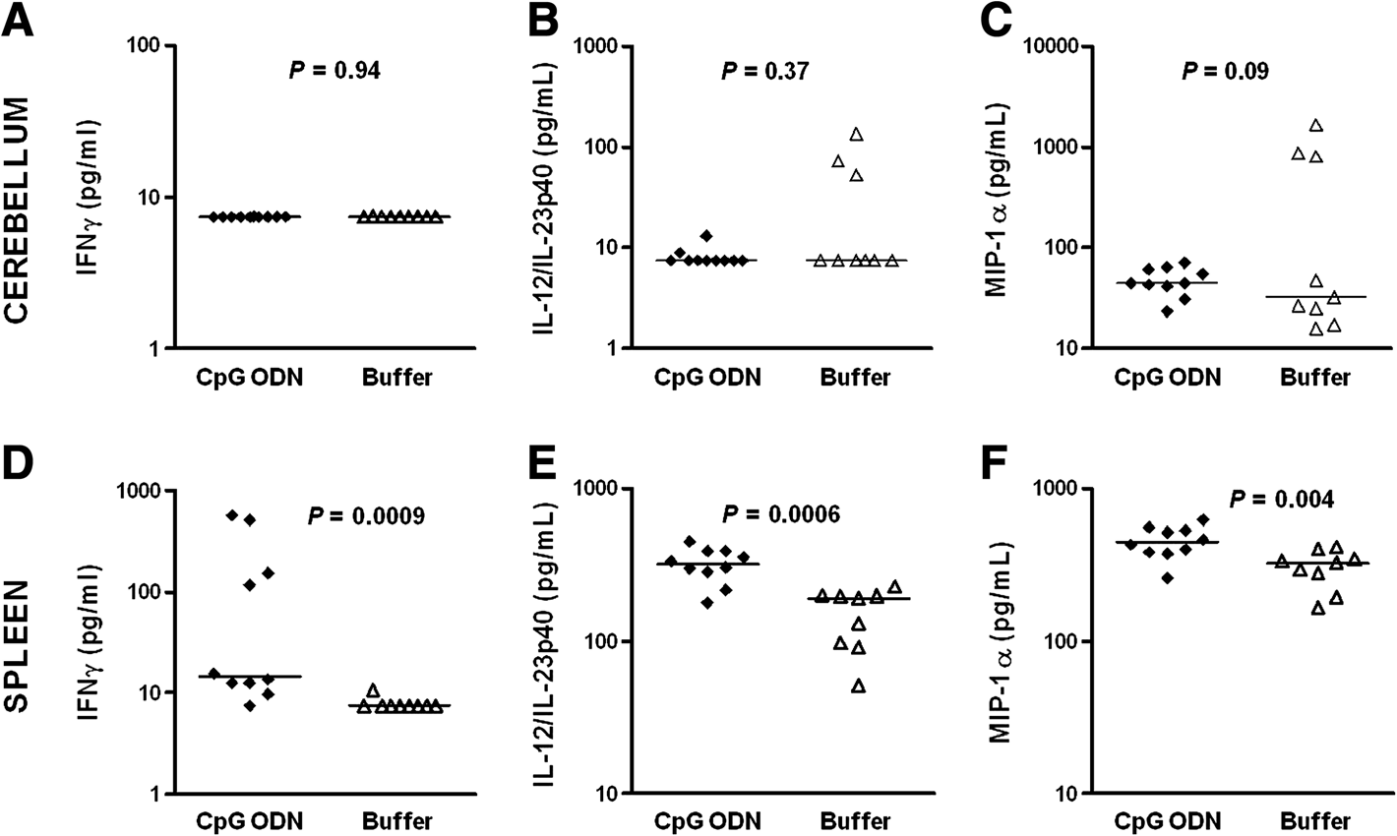
**Prophylaxis with CpG ODN significantly increased IFN-γ, IL-12/IL-23p40, and MIP-1α in spleen homogenates at early infection. (A, D)** IFN-γ, **(B, E)** IL-12/IL-23p40, and **(C, F)** MIP-1α were measured 42 h after intracerebral *E. coli* K1 infection. Each symbol represents an individual neutropenic mouse (*n* = 9-10/group). Horizontal bars indicate median values. Statistical analysis was performed by Mann-Whitney U test.

**Figure 5:**
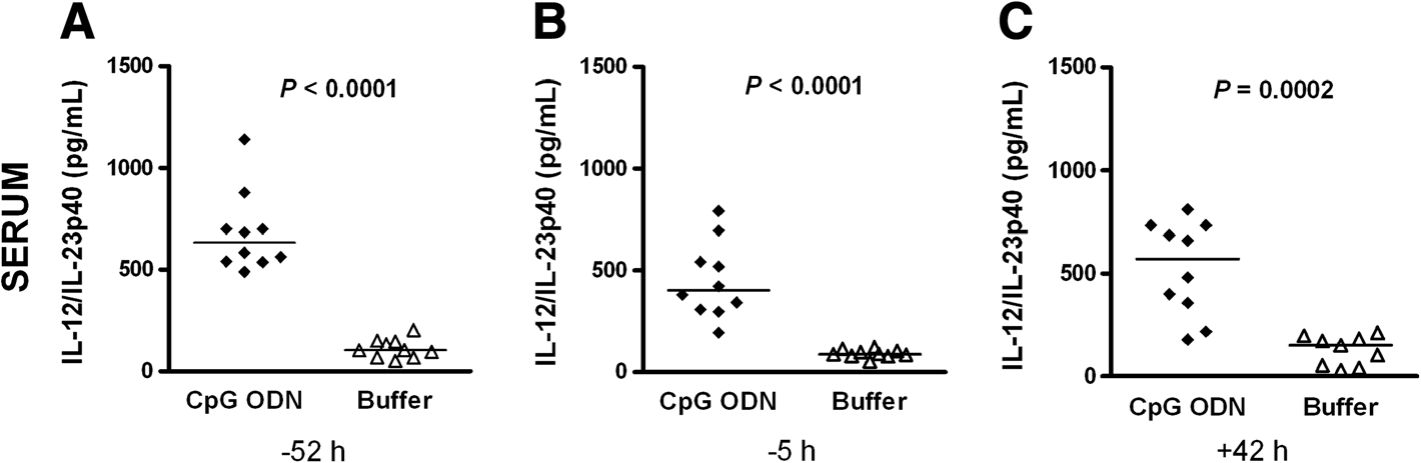
**Protective effect of CpG ODN pre-treatment correlated with high concentrations of IL-12/IL-23p40 in serum.** CpG ODN or buffer was administered 72 h before infection. IL-12/IL-23p40 levels (in pg/mL) were measured at **(A)** 52 h and **(B)** 5 h before infection and **(C)** at the time of sacrifice, that is, 42 h after intracerebral *E. coli* K1 infection (1 × 10^4^ CFU/mouse). Each symbol represents an individual neutropenic mouse (*n* = 9-10/group). Horizontal bars indicate median values. Statistical analysis was performed by Mann-Whitney U test.

We determined the effects of CpG pre-treatment on the production of IL-12 and IFN-γ as Th1-related cytokines [13-15,18-20]. We also measured MIP-1α levels since an increased production was observed after intragastric and intratracheal administration of CpG ODN [26,27]. In neutropenic wt mice (*n* = 9-10/group), IL-12/IL-23p40, IL-12p70, IFN-γ, and MIP-1α levels were measured 42 h after infection (Figures 4 and 5). Concentrations of IL12p70 were low in both groups with median values ranging from 7.5 to 11 pg/mL (data not shown). Animals that were pre-conditioned with CpG ODN showed increased levels of IFN-γ (*P* = 0.0009; Mann-Whitney U test) and IL-12/IL-23p40 (*P* = 0.0006; Mann-Whitney U test) in spleen and higher levels of MIP-1α in both cerebellum (*P* = 0.09; Mann-Whitney U test) and spleen (*P* = 0.004; Mann- Whitney U test) compared to buffer-treated mice (Figure 4). IFN-γ and IL-12/IL-23p40 concentrations in cerebellum homogenates were similar in both groups. A four- to six-fold increase of serum IL-12/IL-23p40 levels was found after the administration of a single ip injection of 100 μg CpG ODN in mice at 52 and 5 h before infection (Figures 5A and B). The increased IL-12/IL-23p40 levels in serum of CpG-treated mice persisted until 42 h (Figure 5C).

**Figure 6:**
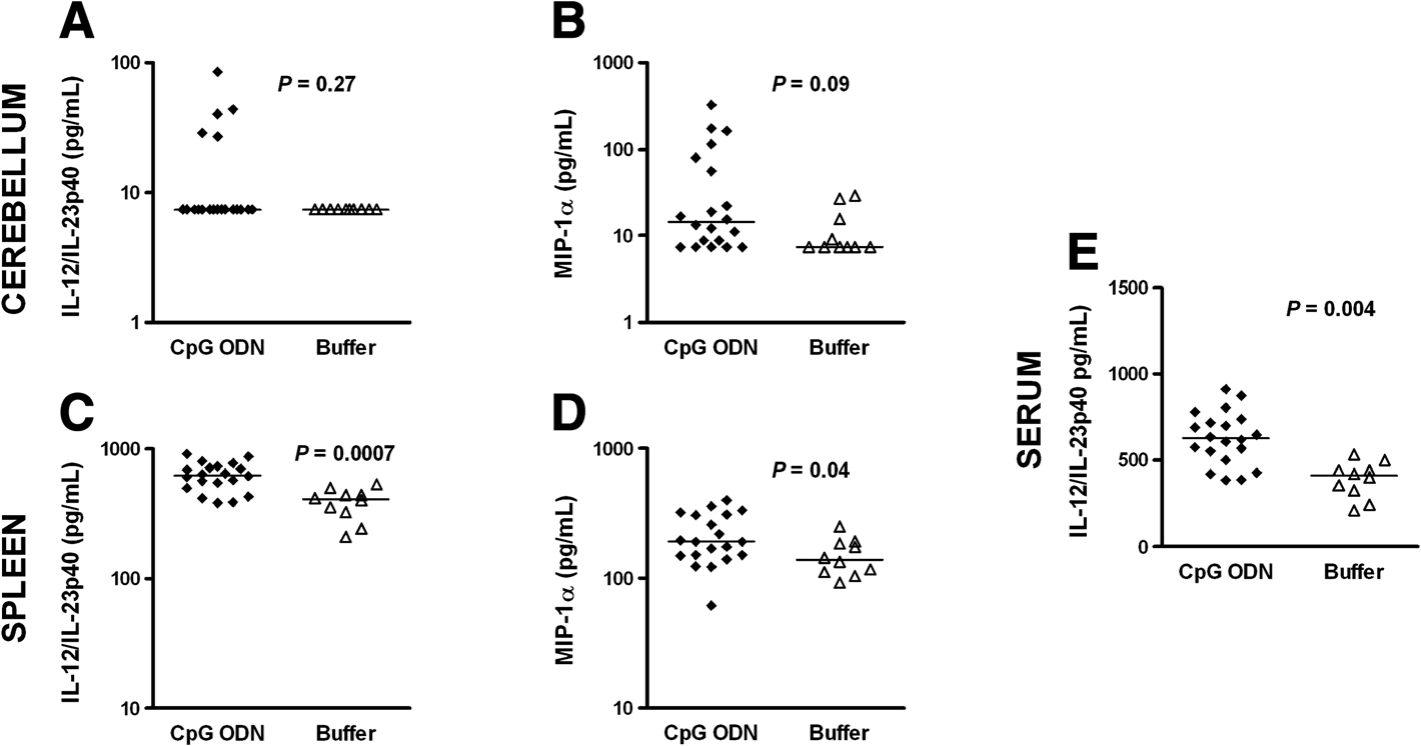
**Survival upon CpG ODN pre-treatment correlated with high levels of IL-12/IL-23p40 and MIP-1α.** Cyto-/chemokine concentrations in **(A, B)** cerebellar and **(C, D)** spleen homogenates and **(E)** serum from CpG- (*n* = 20) and buffer-treated (n = 10) mice were measured 14 days after intracerebral *E. coli* K1 infection. Each symbol represents an individual neutropenic mouse. Horizontal bars indicate median values. Statistical analysis was performed by Mann-Whitney U test.

Fourteen days after infection (Figure 6), surviving neutropenic wt animals pre-conditioned with CpG ODN (*n* = 20) showed higher levels of MIP-1α in both cerebellum (*P* = 0.09; Mann-Whitney U test) and spleen (*P* = 0.04; Mann-Whitney U test) and increased levels of IL-12/IL-23p40 in spleen (*P* = 0.0007; Mann-Whitney U test) and serum (*P* = 0.004; Mann- Whitney U test) compared to buffer-treated mice (*n* = 10). Levels of IL-12/IL-23p40 in cerebellum homogenates were comparable in both groups. To examine whether increased levels of cyto-/chemokines correlated with positive bacterial cultures in cerebellum homogenates of CpG-treated mice (*n* = 20 mice) that survived 2 weeks after infection, the Spearman correlation rank test was performed. The correlation coefficients (r_s_) were +0.08 (*P* = 0.73) for IL-12/IL-23p40 and -0.23 (*P* = 0.33) for MIP-1α concentrations.

### Characterization of the meningeal inflammation after *E. coli* K1 challenge

**Figure 7:**
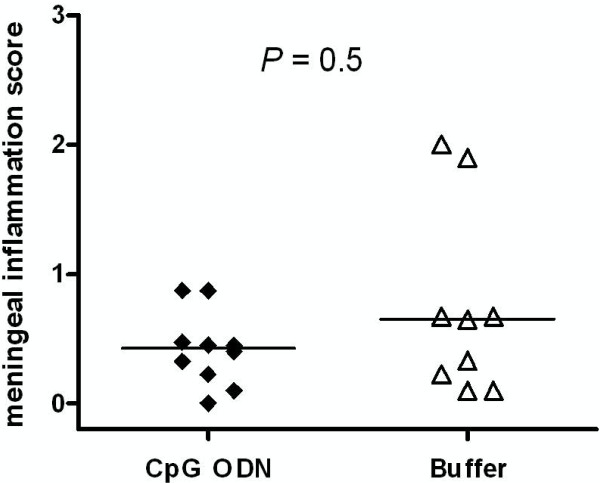
**Meningeal infiltration score was similar in CpG- and buffer-treated animals.** Meningeal infiltration score was calculated in chloroacetate esterase-stained brain sections of CpG- and buffer-treated neutropenic wt mice which were sacrificed 42 h after induction of *E. coli* meninigoencephalitis. Each symbol represents an individual mouse. Bars indicate median values. Statistical analysis was performed by Mann-Whitney U test.

Meningeal inflammation scores from CAE stainings of neutropenic wild-type animals which were sacrificed 42 h after infection are presented in Figure 7. CAE staining confirmed the low amount of infiltrating leukocytes as previously reported [22] and showed no differences between scores in CpG- and buffer-treated animals (median 0.43 (0.16/0.68) *versus* 0.65 (0.16/1.29), *P* = 0.5).

## Discussion

The concept of increasing the resistance against several bacterial infections upon systemic stimulation with one immune activator has been pursued for decades, with mixed success. In rodent models, CpG ODN convey protection against a variety of bloodstream and other extracerebral infections induced by intraperitoneal injection of *Francisella tularensis*[14], *Listeria monocytogenes*[17] and *Burkholderia pseudomallei*[20], oral infection of neonates with *Cryptosporidium parvum*[13], intranasal/intraperitoneal injection of *Yersinia pestis*[16], intratracheal challenge with *Cryptococcus neoformans*[18], against experimental malaria elicited by intravenous injection of *Plasmodium yoelii*[15] and polymicrobial sepsis induced by the colon ascendens stent peritonitis procedure [19].

In the present study, we used mice lacking CD11b^+^Ly6G^+^Ly6C^int^ neutrophils as a model of the immunocompromised host to investigate the capacity of CpG ODN to strengthen the local immune response for a successful elimination of bacteria injected directly into the brain. We were able to show for the first time that systemic immunostimulation with CpG ODN not only protects against systemic bacterial infections but also against an intracerebral challenge with *E. coli*. The CpG motif evaluated in this work stimulated microglial cells and increased their ability to phagoytose and destroy *E. coli* strains intracellularly [11]. Prophylactic intraperitoneal administration of 100 μg of this CpG motif 3 days before intracerebral inoculation of *E. coli* increased the number of neutropenic mice surviving the infection. By observing the time course of the clinical symptoms we assumed that CpG administration increased the local resistance of the brain against infection: only animals which did not develop clinical symptoms (clinical score >1, severe lethargy), survived. Once the infection had spread and caused clinical symptoms, the ultimate outcome was fatal. Prophylaxis with CpG ODN did not prolong the interval from infection to death (median: 43 h for CpG- and buffer-treated neutropenic mice) suggesting a moderate contribution of the systemic inflammatory response which mainly influences the later course of the infection. The lack of protection in TLR9-deficient neutropenic mice strongly suggests that CpG ODN mediated protection by stimulation of TLR9 and not by any other non-specific immunomodulatory effect. Among the animals that survived 14 days after infection, 45% of the CpG-treated mice showed positive bacterial cultures in cerebellum compared to 10% of buffer-treated animals (*P* = 0.08; Mann-Whitney U test). No mouse died later than 6 days after infection. To confirm that CpG-treated animals which were alive after 14 days were able to completely control bacterial growth, one of the survival experiments was extended for a period of 2 months. After 2 months, all surviving CpG pre-conditioned animals had cleared bacteria from brain and spleen. In 2-week survival studies using immunocompetent wt mice, CpG ODN pre-treatment reduced the mortality of the buffer group by only 15% (*P* = 0.2) with no positive cultures in homogenates at the end of the experiment.

In correlation with the enhanced survival, CpG ODN administration significantly reduced bacterial loads in spleen homogenates, an indicator of bacteremia, 42 h after infection. Similarly, bacterial burdens in cerebellum were signicantly lower in mice pre-conditioned with CpG ODN compared to buffer-treated neutropenic animals. However, the meningeal inflammation scores were comparable in both groups. To identify the possible mechanisms behind the CpG-induced protection in our neutropenic mice, we investigated the pattern of cytokine/chemokine production. Because resistance to infections upon CpG ODN pre-conditioning has been related to the promotion of T helper 1 (Th1) responses [13-15,18-20], we focused on IL12 and IFN-γ. IL-12p40 is produced primarily by activated inflammatory cells including monocytes/macrophages, neutrophils, microglia, and dendritic cells (DCs) in response to pathogens mediated by PRRs such as TLRs [28]. IL-12p40 associates with the p35 chain to form IL-12p70, but also with a p19 chain to form IL-23 [29]. In the present study, administration of CpG ODN caused a six-fold increase in serum IL-12/IL-23p40 levels at 52 h before infection. The elevated levels of IL-12/IL-23p40 in serum persisted for at least 17 days after CpG ODN prophylaxis (median: 313 pg/mL in CpG ODN *vs.* 192 pg/mL in buffer-treated mice at 14 days after infection). Correspondingly, CpG-treated animals showed an increased concentration of IL-12/IL-23p40 in spleen homogenates at 42 h, and these high levels were maintained until 14 days after infection. CpG-preconditioned animals showed increased levels of IFN-γ in spleen homogenates compared to the buffer group at 42 h after infection. CpG DNA induces the production of IL-12 that promotes IFN-γ secretion by NK cells [30]. We did not observe differences in the number of NK cells between the CpG and buffer-treated groups at 42 h after infection.

Pre-treatment with CpG ODN induced the recruitment of leukocytes to lung tissue and gastric mucosa in healthy and infected mice [16,27]. In the absence of functional CD11b^+^Ly6G^+^Ly6C^int^ neutrophils, our CpG-treated mice controlled bacterial growth by recruiting higher percentages of Ly6C^high^CCR2^+^ monocytes in both spleen and brain than buffer-treated animals. CpG pre-conditioning also decreased the percentage of CD45^+^CD4^+^CD3^+^CD25^+^FoxP3^+^ regulatory T cells in spleen and brain 42 h after infection. Similarly, during acute infection with *Toxoplasma gondii* and *Listeria monocytogenes* a transient loss of Treg cells caused by IL-2 insufficiency was essential for initiation of Th1 responses and host protection against infection [31]. Furthermore, CpG ODN 2006 downregulated the proportion of Treg cells in peripheral blood mononuclear cells from patients with non-small cell lung cancer [32].

Chemokines are key players in leukocyte recruitment by endothelial cells upon infection. In patients with bacterial meningitis, CSF levels of chemokines including MIP-1α are elevated [33]. Chemokines can be released into the CSF by different cell populations, including microglial cells, resident macrophages and migrating leukocytes depending on the stage of disease [34]. MIP-1α release in human brain microvessel endothelial cells is negligible under physiological conditions but can be upregulated after stimulation with IL-1β or LPS [35]. We found higher levels of MIP-1α in both cerebellum and spleen homogenates of CpG-treated infected animals compared to infected control mice. Peak MIP-1α spleen concentrations were found at 42 h but the levels were elevated until 14 days after infection. We recently reported a strong correlation between *E. coli* K1 loads and increased levels of IL-1β, IL-6, KC (rodent homologue of growth-related oncogene-α/CXCL1), and macrophage inflammatory protein 2 (MIP-2/CXCL2) in cerebellum homogenates of untreated neutropenic wt animals [22]. In the current work, we did not find a significant correlation between the bacterial burdens and the levels of MIP-1α and IL-12/IL-23p40 in cerebellum homogenates of CpG-treated mice that survived 2 weeks after *E. coli* challenge.

The administration of CpG ODN caused weight loss even in uninfected mice. This weight loss was absent in neutropenic TLR9-deficient mice, suggesting that it corresponds to a CpG ODN-induced sickness behavior [25] which has been observed in animal and clinical studies [36-38]. *In vitro*, exposure of co-cultures of neurons with microglial cells to CpG ODN caused neuronal injury, which often started with the attack of microglial cells on axons [39]. In this study, CpG ODN strongly increased survival in spite of the initial weight loss induced by the CpG ODN treatment.

## Conclusions

An intraperitoneal injection of CpG ODN protected neutropenic mice against intracerebral infection with *E. coli* K1 by recruiting higher amounts of inflammatory monocytes into the CNS, decreasing bacterial burdens in the cerebellum and reducing the degree of bacteremia. Therefore, CpG ODN activated the innate immune response not only by influencing the activity of phagocytes of the peritoneum and with direct contact to the circulation, but also those situated in deep compartments such as the CNS. By this way, the systemic administration of CpG ODN may help to prevent CNS infections in immunocompromised individuals even after direct inoculation of bacteria into the intracranial compartments which can occur with open head trauma and after surgery, including placement of an external ventricular drain. Supporting our findings, Marabelle *et al.* recently reported that the addition of CpG to low doses of anti-CTLA-4 and anti-OX40 (two antibodies directed against surface markers of tumor-specific Treg cells) injected locally in a peripheral tumor site in the rat expanded anti-tumor responses to distant tumor sites including the brain [40]. The use of CpG DNA in vaccinations offers several advantages [41]: (1) it can be synthesized with high purity; (2) CpG motifs can exert their immunostimulatory effects either as part of a vaccine or when delivered alone; (3) the presence of CpG motifs contributes to DNA vaccination success rates [42,43]; and (4) Th1 adjuvant properties of CpG DNA enable successful immunization in neonates who are difficult to immunize because of their immature innate immune system [44]. In accordance with this concept, Soogard and collaborators reported the improvement of the immunogenicity of the 7-valent pneumococcal conjugated vaccine in HIV-infected adults by the addition of a variant of CpG (CPG 7909, Coley Pharmaceutical Group) [45]. For these reasons, we suggest conducting a clinical trial with CpG ODN in immunocompromised patients with a high risk of CNS infections.

## Abbreviations

CFU: Colony forming units; CNS: Central nervous system; IFN-γ: Interferon gamma; KC: Rodent homologue of growth-related oncogene-α/CXCL1; MIP-1α: Macrophage inflammatory protein 1 alpha; MIP-2/CXCL2: Macrophage inflammatory protein 2; mAb: Monoclonal antibody; ODN: Oligodeoxynucleotides; PAMPs: Pathogen-associated molecular patterns; PRR: Pattern recognition receptor; PBS: Phosphate buffer solution; Th1: T helper 1; TLRs: Toll-like receptors.

## Competing interests

The authors declare that they have no competing interests.

## Authors’ contributions

SRi and RN designed the study, analyzed the data, interpreted the results, and prepared the manuscript. TM, MO, SRe, and HJ performed experiments and acquired the data. SN analyzed the FACs data and discussed the manuscript. UKH reviewed and discussed the manuscript. All authors have read and approved the final version of the manuscript.

## References

[B1] Ram S Lewis LA Rice PA Infections of people with complement deficiencies and patients who have undergone splenectomy Clin Microbiol Rev 2010 23 740 780 10.1128/CMR.00048-09 20930072 PMC2952982

[B2] Sakran W Levin C Kenes Y Colodner R Koren A Clinical spectrum of serious bacterial infections among splenectomized patients with hemoglobinopathies in Israel: a 37-year follow-up study Infection 2012 40 35 39 10.1007/s15010-011-0178-5 21866338

[B3] Escudier E Cordonnier C Poirier J Infections of the central nervous system in malignant hemopathies Rev Neurol (Paris) 1986 142 116 125 3726388

[B4] Kim KS Strategy of *Escherichia coli* for crossing the blood–brain barrier J Infect Dis 2002 186 S220 S224 10.1086/344284 12424701

[B5] Lu CH Chang WN Chuang YC Chang HW Gram-negative bacillary meningitis in adult post-neurosurgical patients Surg Neurol 1999 52 438 443 10.1016/S0090-3019(99)00129-9 10595761

[B6] Yang TM Lu CH Huang CR Tsai HH Tsai NW Lee PY Chien CC Chang WN Clinical characteristics of adult *Escherichia coli* meningitis Jpn J Infect Dis 2005 58 168 170 15973009

[B7] Liu WM van der Zeijst BA Boog CJ Soethout EC Aging and impaired immunity to influenza viruses: implications for vaccine development Hum Vaccin 2011 7 94 98 10.4161/hv.7.0.14568 21301210

[B8] Klinman DM Yi AK Beaucage SL Conover JS Krieg AM CpG motifs present in bacteria DNA rapidly induce lymphocytes to secrete interleukin 6, interleukin 12, and interferon gamma Proc Natl Acad Sci U S A 1996 93 2879 2883 10.1073/pnas.93.7.2879 8610135 PMC39727

[B9] Weiner GJ The immunobiology and clinical potential of immunostimulatory CpG oligodeoxynucleotides J Leukoc Biol 2000 68 455 463 11037965

[B10] Hemmi H Takeuchi O Kawai T Kaisho T Sato S Sanjo H Matsumoto M Hoshino K Wagner H Takeda K Akira S Toll-like receptor recognizes bacterial DNA Nature 2000 408 740 745 10.1038/35047123 11130078

[B11] Ribes S Ebert S Czesnik D Regen T Zeug A Bukowski S Mildner A Eiffert H Hanisch UK Hammerschmidt S Nau R Toll-like receptor prestimulation increases phagocytosis of *Escherichia coli* DH5alpha and *Escherichia coli* K1 strains by murine microglial cells Infect Immun 2009 77 557 564 10.1128/IAI.00903-08 18981243 PMC2612236

[B12] Ribes S Ebert S Regen T Agarwal A Tauber SC Czesnik D Spreer A Bunkowski S Eiffert H Hanisch UK Hammerschmidt S Nau R Toll-like receptor stimulation enhances phagocytosis and intracellular killing of nonencapsulated and encapsulated *Streptococcus pneumoniae* by murine microglia Infect Immun 2010 78 865 871 10.1128/IAI.01110-09 19933834 PMC2812218

[B13] Barrier M Lacroix-Lamandé S Mancassola R Auray G Bernardet N Chaussé AM Uematsu S Akira S Laurent F Oral and intraperitoneal administation of phosphorothioate oligodeoxynucleotides leads to control of *Cryptosporidium parvum* infection in neonatal mice J Infect Dis 2006 193 1400 1407 10.1086/503748 16619188

[B14] Elkins KL Rhinehart-Jones TR Stibitz S Conover JS Klinman DM Bacterial DNA containing CpG motifs stimulates lymphocyte-dependent protection of mice against lethal infection with intracellular bacteria J Immunol 1999 162 2291 2298 9973506

[B15] Gramzinski RA Doolan DL Sedegah M Davis HL Krieg AM Hoffman SL Interleukin-12- and gamma interferon-dependent protection against malaria conferred by CpG oligodeoxynucleotide in mice Infect Immun 2001 69 1643 1649 10.1128/IAI.69.3.1643-1649.2001 11179339 PMC98068

[B16] Hickey AJ Lin JS Kummer LW Szaba FM Duso DK Tighe M Parent MA Smiley ST Intranasal prophylaxis with CpG oligodeoxynucleotide can protect against *Yersinia pestis* infection Infect Immun 2013 81 2123 2132 10.1128/IAI.00316-13 23545300 PMC3676034

[B17] Krieg AM Love HL Yi AK Harty JT CpG DNA induces sustained IL-12 expression *in vivo* and resistance to *Listeria monocytogenes* challenge J Immunol 1998 161 2428 2434 9725240

[B18] Miyagi K Kawakami K Kinjo Y Uezu K Kinjo T Nakamura K Saito A CpG oligodeoxynucleotides promote the host protective response against infection with *Cryptococcus neoformans* through induction of interferon-gamma production by CD4^+^ T cells Clin Exp Immunol 2005 140 220 229 10.1111/j.1365-2249.2005.02772.x 15807845 PMC1809361

[B19] Weighardt H Feterowski C Veit M Rump M Wagner H Holzmann B Increased resistance against acute polymicrobial sepsis in mice challenged with immunostimulatory CpG oligodeoxynucleotides is related to an enhanced innate effector cell response J Immunol 2000 165 4537 4543 11035094 10.4049/jimmunol.165.8.4537

[B20] Wongratanacheewin S Kespichayawattana W Intachote P Pichyangkul S Sermswan RW Krieg AM Sirisinha S Immunostimulatory CpG oligodeoxynucleotide confers protection in a murine model of infection with *Burkholderia pseudomallei* Infect Immun 2004 72 4494 4502 10.1128/IAI.72.8.4494-4502.2004 15271908 PMC470634

[B21] Gerber J Raivich G Wellmer A Noeske C Kunst T Werner A Brück W Nau R A mouse model of *Streptococcus pneumoniae* meningitis mimicking several features of human disease Acta Neuropathol 2001 101 499 508 11484822 10.1007/s004010000326

[B22] Ribes S Regen T Meister T Tauber SC Schütze S Mildner A Mack M Hanisch UK Nau R Resistance of the brain to *Escherichia coli* K1 Infection depends on MyD88 signaling and the contribution of neutrophils and monocytes Infect Immun 2013 81 1810 1819 10.1128/IAI.01349-12 23478323 PMC3648016

[B23] Hu W Nessler S Hemmer B Eagar TN Kane LP Leliveld SR Müller-Schiffmann A Gocke AR Lovett-Racke A Ben LH Hussain RZ Breil A Elliott JL Puttaparthi K Cravens PD Singh MP Petsch B Stitz L Racke MK Korth C Stüve O Pharmacological prion protein silencing accelerates central nervous system autoimmune disease via T cell receptor signalling Brain 2010 133 375 388 10.1093/brain/awp298 20145049 PMC2822628

[B24] Scheffel J Regen T Van Rossum D Seifert S Ribes S Nau R Parsa R Harris RA Boddeke HW Chuang HN Pukrop T Wessels JT Jürgens T Merkler D Brück W Schnaars M Simons M Kettenmann H Hanisch UK Toll-like receptor activation reveals developmental reorganization and unmasks responder subsets of microglia Glia 2012 60 1930 1943 10.1002/glia.22409 22911652

[B25] McCusker RH Kelley KW Immune-neural connections: how the immune system’s response to infectious agents influences behavior J Exp Biol 2013 216 84 98 10.1242/jeb.073411 23225871 PMC3515033

[B26] Raghavan S Nyström J Fredriksson M Holmgren J Harandi AM Orally administered CpG oligodeoxynucleotide induces production of CXC and CC chemokines in the gastric mucosa and suppresses bacterial colonization in a mouse model of *Helicobacter pylori* infection Infect Immun 2003 71 7014 7022 10.1128/IAI.71.12.7014-7022.2003 14638791 PMC308895

[B27] Tasaka S Kamata H Miyamoto K Nakano Y Shinoda H Kimizuka Y Fujiwara H Hasegawa N Fujishima S Miyasho T Ishizaka A Intratracheal synthetic CpG oligodeoxynucleotide causes acute lung injury with systemic inflammatory response Respir Res 2009 10 84 10.1186/1465-9921-10-84 19772669 PMC2761862

[B28] Trinchieri G Pflanz S Kastelein RA The IL-12 family of heterodimeric cytokines: new players in the regulation of T cell responses Immunity 2003 19 641 644 10.1016/S1074-7613(03)00296-6 14614851

[B29] Oppmann B Lesley R Blom B Timans JC Xu Y Hunte B Vega F Yu N Wang J Singh K Zonin F Vaisberg E Churakova T Liu M Gorman D Wagner J Zurawski S Liu Y Abrams JS Moore KW Rennick D de Waal-Malefyt R Hannum C Bazan JF Kastelein RA Novel p19 protein engages IL-12p40 to form a cytokine, IL-23, with biological activities similar as well as distinct from IL-12 Immunity 2000 13 715 720 10.1016/S1074-7613(00)00070-4 11114383

[B30] Chace JH Hooker NA Mildenstein KL Krieg AM Cowdery JS Bacterial DNA-induced NK cell IFN-gamma production is dependent on macrophage secretion of IL-12 Clin Immunol Immunopathol 1997 84 185 193 10.1006/clin.1997.4380 9245551

[B31] Benson A Murray S Divakar P Burnaevskiy N Pifer R Forman J Yarovinsky F Microbial infection-induced expansion of effector T cells overcomes the suppressive effects of regulatory T cells via an IL-2 deprivation mechanism J Immunol 2012 188 800 810 10.4049/jimmunol.1100769 22147768 PMC3253229

[B32] Wang YY He XY Cai YY Wang ZJ Lu SH The variation of CD4^+^CD25^+^ regulatory T cells in the periphery blood and tumor microenvironment of non-small cell lung cancer patients and the downregulation effects induced by CpG ODN Target Oncol 2011 6 147 154 10.1007/s11523-011-0182-9 21611754

[B33] Spanaus KS Nadal D Pfister HW Seebach J Widmer U Frei K Gloor S Fontana A C-X-C and C-C chemokines are expressed in the cerebrospinal fluid in bacterial meningitis and mediate chemotactic activity on peripheral blood-derived polymorphonuclear and mononuclear cells *in vitro* J Immunol 1997 158 1956 1964 9029138

[B34] Galanakis E Di Cello F Paul-Satyaseela M Kim KS *Escherichia coli* K1 induces IL-8 expression in human brain microvascular endothelial cells Eur Cytokine Netw 2006 17 260 265 17353159

[B35] Chui R Dorovini-Zis K Regulation of CCL2 and CCL3 expression in human brain endothelial cells by cytokines and lipopolysaccharide J Neuroinflammation 2010 7 1 10.1186/1742-2094-7-1 20047691 PMC2819252

[B36] Carpentier A Laigle-Donadey F Zohar S Capelle L Behin A Tibi A Martin-Duverneuil N Sanson M Lacomblez L Taillibert S Puybasset L Van Effenterre R Delattre JY Carpentier AF Phase 1 trial of a CpG oligodeoxynucleotide for patients with recurrent glioblastoma Neuro Oncol 2006 8 60 66 10.1215/S1522851705000475 16443949 PMC1871923

[B37] Carpentier A Metellus P Ursu R Zohar S Lafitte F Barrié M Meng Y Richard M Parizot C Laigle-Donadey F Gorochov G Psimaras D Sanson M Tibi A Chinot O Carpentier AF Intracerebral administration of CpG oligonucleotide for patients with recurrent glioblastoma: a phase II study Neuro Oncol 2010 12 401 408 10.1093/neuonc/nop047 20308317 PMC2940609

[B38] Kozak W Wrotek S Kozak A Pyrogenicity of CpG-DNA in mice: role of interleukin-6, cyclooxygenases, and nuclear factor-kappaB Am J Physiol Regul Integr Comp Physiol 2006 290 R871 R880 16293680 10.1152/ajpregu.00408.2005

[B39] Iliev AI Stringaris AK Nau R Neumann H Neuronal injury mediated via stimulation of microglial Toll-like receptor 9 (TLR9) FASEB J 2004 18 412 414 14688201 10.1096/fj.03-0670fje

[B40] Marabelle A Kohrt H Sagiv-Barfi I Ajami B Axtell RC Zhou G Rajapaksa R Green MR Torchia J Brody J Luong R Rosenblum MD Steinman L Levitsky HI Tse V Levy R Depleting tumor-specific Tregs at a single site eradicates disseminated tumors J Clin Invest 2013 123 2447 2463 10.1172/JCI64859 23728179 PMC3668834

[B41] Dalpke A Zimmermann S Heeg K CpG DNA in the prevention and treatment of infections BioDrugs 2002 16 419 431 10.2165/00063030-200216060-00003 12463765

[B42] Klinman DM Yamshchikov G Ishigatsubo Y Contribution of CpG motifs to the immunogenicity of DNA vaccines J Immunol 1997 158 3635 3639 9103425

[B43] Sato Y Roman M Tighe H Lee D Corr M Nguyen MD Silverman GJ Lotz M Carson DA Raz E Immunostimulatory DNA sequences necessary for effective intradermal gene immunization Science 1996 273 352 354 10.1126/science.273.5273.352 8662521

[B44] Bot A Bona C Genetic immunization of neonates Microbes Infect 2002 4 511 520 10.1016/S1286-4579(02)01566-6 11932202

[B45] Søgaard OS Lohse N Harboe ZB Offersen R Bukh AR Davis HL Schønheyder HC Østergaard L Improving the immunogenicity of pneumococcal conjugate vaccine in HIV-infected adults with a toll-like receptor 9 agonist adjuvant: a randomized, controlled trial Clin Infect Dis 2010 51 42 50 10.1086/653112 20504165

